# Short-Loop Recycling of Nd-Fe-B Permanent Magnets: A Sustainable Solution for the RE_2_Fe_14_B Matrix Phase Recovery

**DOI:** 10.3390/ma16196565

**Published:** 2023-10-05

**Authors:** Amit Mishra, Sina Khoshsima, Tomaž Tomše, Benjamin Podmiljšak, Sašo Šturm, Carlo Burkhardt, Kristina Žužek

**Affiliations:** 1 Department of Nanostructured Materials, Jožef Stefan Institute, Jamova Cesta 39, 1000 Ljubljana, Slovenia; sina.khoshsima@ijs.si (S.K.); tomaz.tomse@ijs.si (T.T.); beno.podmiljsak@ijs.si (B.P.); saso.sturm@ijs.si (S.Š.); tina.zuzek@ijs.si (K.Ž.); 2 International Postgraduate School, Jožef Stefan Institute, Jamova Cesta 39, 1000 Ljubljana, Slovenia; 3 Institute for Precious and Technology Metals, Pforzheim University, 75175 Pforzheim, Germany; carlo.burkhardt@hs-pforzheim.de

**Keywords:** recycling, leaching, Nd-Fe-B magnets, hydrogen decrepitation, rare earth elements, grain boundary phase, (Nd, Pr)_2_Fe_14_B grains

## Abstract

The green transition initiatives and exploitation of renewable energy sources require the sustainable development of rare earth (RE)-based permanent magnets prominent technologies like wind turbine generators and electric vehicles. The recycling of RE-based permanent magnets is necessary for the future supply of critical rare-earth elements. The short-loop recycling strategies to directly reprocess Nd-Fe-B magnet waste are economically attractive and practical alternatives to conventional hydro- and pyrometallurgical processes. This study focuses on the development of a procedure to extract the (Nd, Pr)_2_Fe_14_B hard-magnetic phase from sintered Nd-Fe-B magnets. The extraction is achieved through preferential chemical leaching of the secondary, RE-rich phases using 1 M citric acid. Before the acid treatment, the magnets were pulverized through hydrogen decrepitation (HD) to increase the material’s surface-to-volume ratio. The as-pulverized Nd-Fe-B powder was subsequently exposed to a 1 M citric acid solution. The effect of leaching time (5–120 min) on the phase composition and magnetic properties was studied. The results of the microstructural (SEM) and compositional (ICP-MS) analyses and the study of thermal degassing profiles revealed that the RE-rich phase is preferentially leached within 5–15 min of reaction time. Leaching of the secondary phases from the magnet’s multi-phase microstructure is governed by the negative electrochemical potential of Nd and Pr. The extraction of (Nd, Pr)_2_Fe_14_B grains by the proposed acid leaching approach is compatible with the existing hydrogen processing of magnetic scrap (HPMS) technologies. The use of mild organic acid as a leaching medium makes the leaching process environmentally friendly, as the leaching medium can be easily neutralized after the reaction is completed.

## 1. Introduction

Among the rare-earth-based permanent magnets, the Nd-Fe-B magnets offer the highest maximum energy products (up to 450 kJ/m^3^) [1]. This opens up their prospects in numerous applications, e.g., traction motors of electric vehicles (EVs) and wind turbine generators, consumer electronics, and home appliances [2,3,4]. The magnets’ life span depends upon their application and can be as short as 2–3 years (consumer electronics) or 20–30 years in the case of wind turbines. Recycling rare earth-based permanent magnets has gained importance due to price rises and the shortage of rare earth (RE) elements and, thus, plays an essential role in the long-term sustainability and reliable future supply of these critical materials [5,6,7,8,9]. Sintered Nd-Fe-B magnets that account for more than half of the magnet market by value [10] contain 31–32 wt.% of REs (mostly Nd), making them an excellent secondary source for RE recovery. Magnet recycling is currently facing many technical challenges, and most recycling techniques are still in the research and development stages. On the one hand, short-loop recycling (direct reprocessing) can be considered eco-friendly and economically justifiable due to its guarantee of zero waste generation and simplified reprocessing steps. The recycling strategy is based on the hydrogen processing of magnetic scrap (HPMS) [11] to pulverize the end-of-life (EoL) magnets, followed by further milling, and finally re-sintering the recycled powders to produce new magnets [12]. On the other hand, long-loop recycling strategies based on hydrometallurgical and pyrometallurgical methods to recover the precious constituting elements from magnet waste encompass complex and expensive procedures. Hydrometallurgical processing is associated with a large amount of chemical consumption and wastewater generation [13], and the pyrometallurgical method is energy-intensive as it works at very high temperatures [14,15].

Nd-Fe-B magnets are based primarily on the RE_2_Fe_14_B hard-magnetic phase, where part of Nd can be substituted with other REs, such as Pr, Dy, or Tb [16,17,18]. The secondary, RE-rich phases, found at the RE_2_Fe_14_B grain boundaries and triple junctions, present 10-12 volume % of sintered Nd-Fe-B magnets. A RE-rich phase readily corrodes upon exposure to air and humidity due to the very negative reduction potentials of REs, i.e., Nd (−2.32 V) and Pr (−2.35 V) [19,20]. The potential for the short-loop recycling of magnets compromised by oxidation is greatly diminished, as the recycled magnets’ performance is inferior to virgin magnets with a carefully tailored chemical composition [3,21]. However, the RE_2_Fe_14_B grains are generally intact after the magnet-containing device has been put out of use. Therefore, they retain their potential to be directly reused as primary building blocks of novel Nd-Fe-B magnets. Recently, the electrochemical recovery of Nd_2_Fe_14_B grains from bulk sintered Nd-Fe-B magnets was realized via the anodic leaching of RE-rich phases, considering the negative reduction potentials of REs [5]. The work highlighted an important correlation between the microstructure and the leaching mechanism: the highly reactive RE-rich phases were leached first, followed by the Nd_2_Fe_14_B grains. In the previous study carried out by our group, the proof of concept on selective leaching of the RE-rich phase was demonstrated on strip cast Nd-Fe-B feedstock powder. It was shown that citric acid is selective towards the leaching of the RE-rich phase compared to the hard magnetic phase for the first time [22]. The outcome of that work was chosen as basis of this work [22]. 

In this paper, we investigate the RE_2_Fe_14_B magnetic phase extraction from sintered Nd-Fe-B magnets by the preferential chemical leaching of the secondary phases using a weak organic acid. At first, sintered Nd-Fe-B magnets were hydrogen-decrepitated, i.e., pulverized, with hydrogen gas at room temperature. The hydrogenation process leads to the formation of RE-rich hydride phases at the triple grain junctions and grain boundaries [23] and the interstitial hydrogen solution in the matrix grains (RE_2_Fe_14_BH_X_) [24]. The hydrogenation process is environmentally friendly just like the floatation, which has been used to upgrade the alloy or mineral surface before leaching [25,26]. The absorption of hydrogen and the corresponding volume expansion of grain lattice results in strain and facilitates crack formation along the grain boundaries [27]. The decrepitated Nd-Fe-B powder with an increased surface-to-volume ratio was then exposed to 1 M citric acid for 5 to 120 min. The effect of the reaction time on the material’s phase composition was studied. The microstructural investigation, thermal degassing profiles, elemental compositional analysis, and magnetic characterization of the leached powder samples revealed that the RE_2_Fe_14_B grains were extracted from the hydrogen-decrepitated Nd-Fe-B magnets without compromising their magnetic performance. The RE-rich phases were preferentially leached within 5-15 min of exposure to citric acid, while longer reaction times led to the continuous leaching of the matrix phase. The RE_2_Fe_14_B grains can be extracted from EOL sintered magnets using this method and can either be resintered to obtain recycled magnets or used for novel magnet making.

## 2. Materials and Methods

### 2.1. Hydrogen Decrepitation of Nd-Fe-B Magnet

Uncoated sintered Nd-Fe-B magnets (10 mm (height) × 20 mm (diameter)) were broken down into two or more small pieces and subsequently pulverized with hydrogen gas (1 bar) at room temperature. The hydrogen decrepitation was performed in a semi-closed tube, with one side having a dual connection to the vacuum pump and hydrogen gas. The process was carried out for 15 h. The powder obtained was labeled as NFB-HD. The chemical composition of the NFB-HD material is given in Section 3.4.

### 2.2. Leaching of Hydrogen-Decrepitated Nd-Fe-B Powder with Citric Acid

The leaching experiments performed at room temperature on the NFB-HD powder were carried out with citric acid (Sigma Aldrich Pvt. Ltd., Darmstadt, Germany), diluted to a concentration of 1 M. The solid-to-liquid ratio was 1:15 (1 g of NFB-HD powder and 15 mL of citric acid). The leaching reaction was accompanied by the evolution of gas bubbles, attributed to the release of H_2_. Six samples were prepared by varying the leaching time: the NFB-HD powder was left in the acid solution for 5, 15, 30, 45, 60, or 120 min. After leaching for the prescribed time, the leachate was slowly and carefully decanted and collected in a separate vial for the subsequent ICP-MS compositional analysis. The leached powder samples were then washed three times with deionized water and ethanol, dried at 60 °C, and stored under an argon atmosphere. The samples were labeled as NFB-X, where X is the corresponding leaching time in min (NFB-5, NFB-15, NFB-30, NFB-45, NFB-60, and NFB-120).

### 2.3. Characterization of Samples

The microstructural and phase analyses of the initial Nd-Fe-B magnet, hydrogen-decrepitated powder, and leached powder samples were carried out with a field emission-gun scanning electron microscope FEG-SEM JEOL JSM-7600F equipped with energy-dispersive X-ray spectroscopy (EDXS) (INCA system, Oxford Instruments, Abingdon, UK) with point-beam analysis. For this purpose, the magnet was embedded in Viscosit resin (Struers Pvt. Ltd., Ballerup, Denmark), while the powders were embedded in Epofix resin inside the EPOVAC vacuum chamber. The SEM samples were polished on MD-DAC and MD-DAP sheets (Struers Pvt. Ltd., Ballerup, Denmark) with 3 and 0.25 µm diamond pastes for a smooth finish and washed with isopropanol. The mass magnetization values of leached powders were measured with a Lakeshore 8600 vibrating-sample magnetometer (VSM) calibrated by Ni ball. Four iterative measurements were carried out on each sample. Compositional analysis of the powders and leachates by ICP/MS was performed by Agilent 7700x Series (Agilent Technologies, Tokyo, Japan) ICP-MS equipped with an ASX-500 autosampler. Degassing experiments were performed on the NFB-HD and leached powder samples to assess the presence of RE-hydride phases qualitatively. The degassing was carried out in a tube furnace (Nabertherm Gmbh, Lilienthal, Germany) attached to a vacuum pump (Pfeiffer Vacuum, Aβlar, Germany). A quantity of 2 g of a powder was heated from room temperature to 700 °C at a heating rate of 2 °C per minute. The change in the pressure concerning the temperature was monitored.

## 3. Results and Discussion

### 3.1. Microstructure of Sintered Nd-Fe-B Magnet

**Figure 1:**
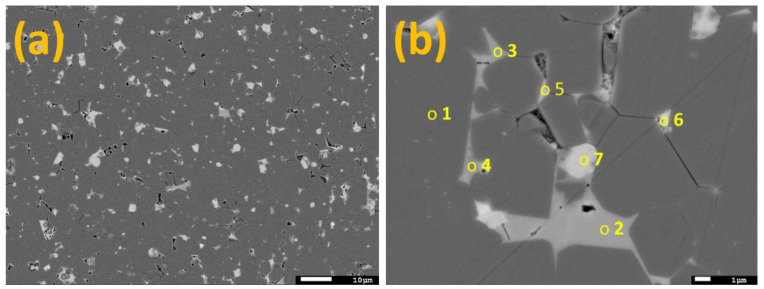
Backscattered SEM images showing the initial sintered Nd-Fe-B magnet microstructure: (**a**) low and (**b**) high magnification with marked locations of EDS local compositional analyses.

Backscattered-electron (BSE) mode SEM images of the Nd-Fe-B magnet are shown in Figure 1. The microstructure is multi-phase, consisting of a grey matrix phase and grains with brighter contrast, as observed through the compositional contrast in the low-magnification image (Figure 1a). Such a microstructure is typical for sintered Nd-Fe-B magnets [28]. EDS microanalysis was performed on the locations marked in the high-magnification images (Figure 1b) to probe the chemical compositions of the respective crystal phases. The results of the analysis are gathered in Table 1. The gray matrix (Site 1) comprises Fe and RE elements Nd and Pr, identifying it as RE_2_Fe_14_B (boron was not quantified). The secondary phases (Sites 2–7) at triple grain pockets appear brighter in contrast due to a higher amount of the REs (28.3, 27.9, 20.0, 49.1, 52.9, and 50.3 at. %) in comparison to the matrix phase (13.4 at. %). As seen in Table 1, the chemical composition of the secondary phases is diverse. As measured via EDS, Sites 2, 3, and 4 contain Ga (6.2, 4.3, and 3.9 at. %, respectively) in addition to REs and Fe. Sites 3 and 4 contain Al (2.0 and 1.5 at. %, respectively), while Sites 5 and 6 contain Co (10.9 and 10.2 at. %, respectively). Ga and Al are added to the alloy to improve the grain boundary wetting and increase the coercivity [29], [30], while Co is added to ensure a higher Curie temperature and better corrosion resistance in magnets [31]. Co can substitute for Fe in the matrix phase [32]; however, the concentration was too low to be quantified for Site 1 with the EDS. Site 7 contains a high oxygen content (46.3 at. %), identifying the analyzed grain as RE-oxide. Note that the exact composition of the bright RE-rich phases cannot be easily assessed with this analysis due to the analytical limitation of the EDS technique, i.e., large interaction volume (≈1 µm^3^ for the analytical conditions used) compared to the grain size. 

### 3.2. Microstructure of NFB-HD and Leached Powder Samples

**Figure 2:**
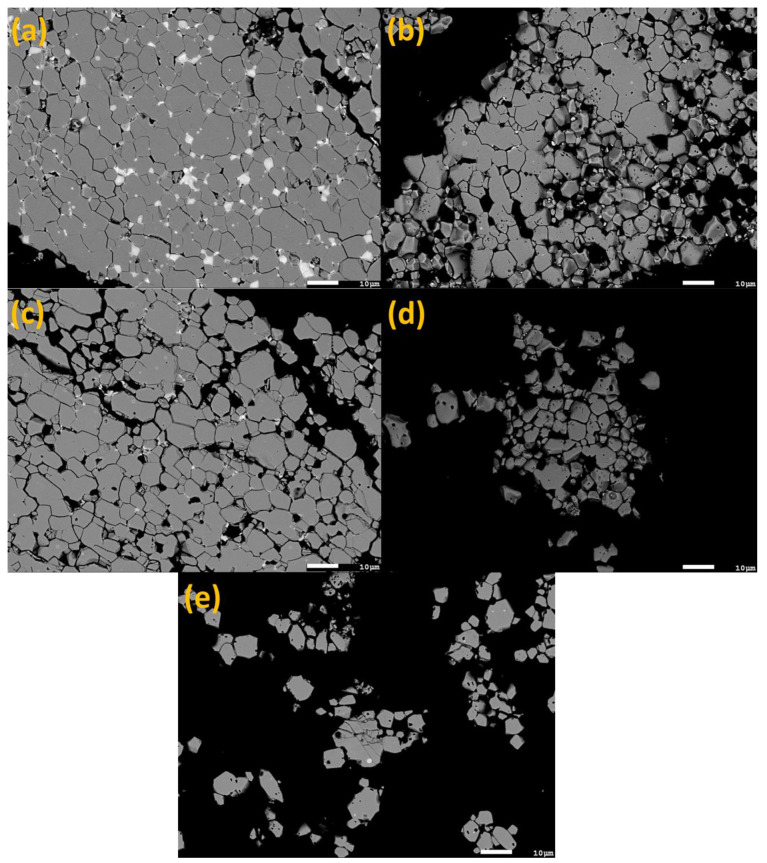
BSE-SEM images of (**a**) NFB-HD, (**b**) NFB-5, (**c**) NFB-15, (**d**) NFB-45, and (**e**) NFB-120 acid-leached samples.

Figure 2a shows the BSE-SEM image of the hydrogen-decrepitated powder (NFB-HD). The powder particle shown in the image is polycrystalline. Its microstructure consists of matrix grains and RE-rich phases displaying dark and bright contrasts, respectively. The microstructures of the NFB-HD powder particle and the initial sintered Nd-Fe-B magnet (Figure 1a) are comparable. In Figure 2a, the cracks and fractures along the grain boundaries, resulting from the expansion of the RE_2_Fe_14_B grains and RE-rich phases following the hydrogen treatment (Section 2.1), are observed. The hydrogen-decrepitation process broke the magnet into a coarse powder with a particle size ranging from approx. 250 to 500 μm, estimated from the SEM images (not shown). Figure 2b–e shows the microstructures of the hydrogen-decrepitated powder after acid leaching for 5, 15, 45, and 120 min. Compared to the non-leached NFB-HD powder, the volume fraction of the secondary RE-rich phases is significantly reduced in the leached samples. After 5 min of leaching time (sample NFB-5), only a few individual small grains displaying a bright contrast can be observed in the BSE-SEM image (Figure 2b). The RE-rich phases appear to almost disappear completely for powder samples obtained after 15 (NFB-15, Figure 2c), 45 (NFB-45, Figure 2d), and 120 min (NFB-120, Figure 2e) of leaching time. This suggests that most of the RE-rich phases were preferentially leached by citric acid. However, the SEM analysis provides only local information on the phase composition and offers a limited spatial resolution. Therefore, additional experiments were performed, as detailed in the following sections, to provide more quantitative information on the leaching efficiency and selectivity towards the RE-rich phases. 

### 3.3. Degassing Profiles

**Figure 3:**
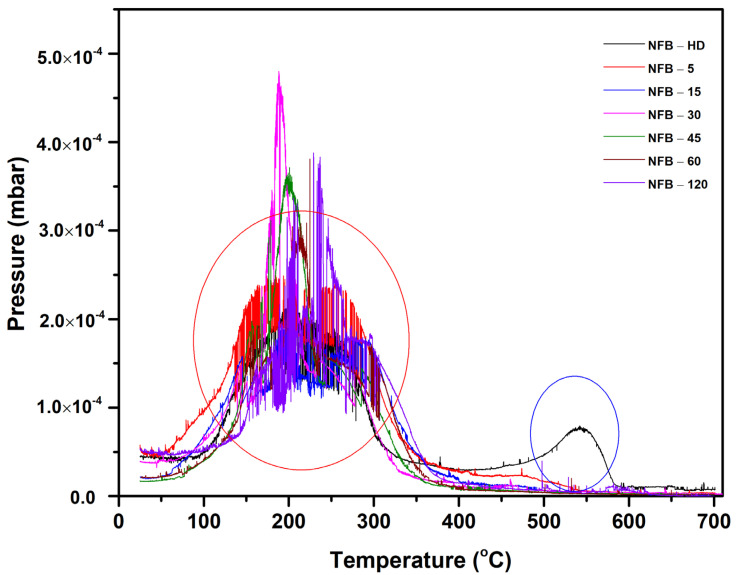
Degassing profiles of the NFB-HD and leached powder samples.

The hydrogen-decrepitated powder (NFB-HD) and leached powder samples (NFB-5, NFB-15, NFB-30, NFB-45, NFB-60, and NFB-120) were heated under vacuum to degas the hydrogen from the materials and study the change in the phase composition upon the acid treatment. Figure 3 shows the evolution of pressure with the temperature during heating (degassing profiles). For all the samples, the first increase in pressure is observed in the temperature range of 100–350 °C (within red circle). For the NFB-HD powder (black curve in Figure 3), the hydrogen desorption occurs in stages as the pressure increases again at higher temperatures, peaking at 550 °C (within blue circle). As is known from the literature, the hydrogen desorption kinetics vary for the different constituting phases (RE_2_Fe_14_BH_x_ and RE-rich hydride phases) [12,33,34]. The low-temperature peak indicates a complete hydrogen desorption from the RE_2_Fe_14_B phase and a partial, i.e., incomplete, desorption from the RE-rich phases, as both desorption events occur at similar temperatures [12]. The high-temperature peak (within blue circle) signifies the complete desorption of hydrogen from the RE-rich phases [12,35]. The two-stage degassing profile observed for the NFB-HD powder agrees with the material’s multi-phase structure. For the NFB-5 sample, the second peak in the 400–600 °C range (within blue circle) is diminished significantly (red curve in Figure 3). For samples leached for 15 min or longer, the second peak is no longer present. The degassing profiles recorded for the leached powder samples, therefore, indicate a reduced fraction of the secondary RE-rich hydride phases, compared to the NFB-HD powder, reaffirming the results of the SEM investigation (Figure 2). Accordingly, the absence of the second peak in the degassing profiles of leached powder samples exposed to 1 M citric acid for 15 min or longer confirms that the resulting powders consist of the RE_2_Fe_14_B phase.

### 3.4. Compositional Analysis of NFB-HD, Leached Powder Samples, and Leachates by ICP-MS Analysis

**Figure 4:**
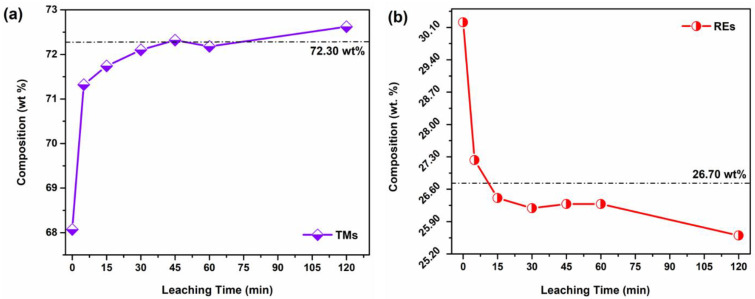
Trends showing variation in the composition of (**a**) TMs (Fe+Co) and (**b**) REs (Nd+Pr) in powder samples obtained after different leaching times.

The elemental compositions of the hydrogen-decrepitated NFB-HD powder and citric-acid-leached powder samples are shown in Table 2. The stoichiometric composition of the RE_2_Fe_14_B phase is added for comparison; it consists of 26.70 wt.% of REs, 72.30 wt.% of transition metals (TMs) (Fe+Co), and 0.99 wt. % of B. The NFB-HD powder contains 30.21 wt.% of REs (Nd+Pr), i.e., 3.5 wt. % access compared to the RE_2_Fe_14_B. The RE content complies with the material’s multi-phase microstructure, i.e., the presence of secondary RE-rich phases (Figure 2a). Such a high RE content is expected for the compositions of sintered Nd-Fe-B magnets to enable the liquid-phase sintering and ensure grain-boundary wetting to decouple the matrix grains in terms of exchange interactions [36]. The concentrations of minor alloying elements, i.e., Cu (0.09 wt.%), Ga (0.22 wt.%), and Al (0.13 wt. %), are comparably low and not addressed in assessing the leaching mechanism. For the powder sample leached for 5 min (NFB-5), the mass fraction of REs is reduced to 27.23 wt.%. Compared to the stoichiometric RE_2_Fe_14_B, the NFB-5 material contains ≈ 0.5 wt.% excess of REs. This agrees with the SEM analysis of the sample (Figure 2b) and its degassing characteristics (Figure 3, Section 3.3.), confirming the presence of RE-rich phases. For samples NFB-15, NFB-30, NFB-45, and NFB-60, their RE content (26.40, 26.19, 26.28, and 26.27 wt.%, respectively) is close to the stochiometric RE_2_Fe_14_B (26.70 wt.%). In short, the near-stoichiometric composition of REs in leached powder samples was obtained within 15 min of leaching time. Contrasting and complementing the reduced RE concentration in the leached powders, and the TM concentration increased from 68.07 wt.% (NFB-HD) to ≈ 72 wt.% for samples leached for 15 min or longer. Figure 4 shows the change in the TM and RE concentrations (Figure 4a,b, respectively) vs. the leaching time. The stoichiometric compositions are marked with dashed lines. The trends were further analyzed to calculate the rates of concentration change for the different leaching time intervals. The change rates are calculated using the expression:

(1) $$r=\frac{wt{\%}_{2}-wt{\%}_{1}}{t_{2}-t_{1}}$$

where *r* is the rate of change at time intervals *t*_1_−*t*_2_, and $wt{\%}_{2}$ and $wt{\%}_{1}$ are mass fractions at times t_2_ and t_1_, respectively. The rate of change in the TM concentration was the highest in the 0–5 min interval: the mass fraction of TMs increased from 68.07 to 71.32 wt%, at a rate of 0.65 wt%/minute. The rate of change then decreased for longer leaching times to 0.042, 0.024, and 0.002 wt%/min for 5–15, 15–30, and 30–60 min time intervals, respectively. Similarly, the rate of change in the RE concentration was highest in the 0–5 min interval at −0.59 wt%/min and dropped to −0.082, −0.014, and −0.006 wt%/min for 5–15, 15–30, and 30–60 min time interval, respectively. The trends, therefore, indicate that the leaching rate of the REs is the highest in the first 5 min of exposure to citric acid, after which the reaction progressively slows down for the longer reaction times. The RE/TM ratio was considered to assess the leaching dynamics further. The stoichiometric RE/TM ratio of the RE_2_Fe_14_B phase equals 0.37. When NFB-HD was leached for 5 min (sample NFB-5), the RE/TM ratio decreased from 0.44 to 0.39. After 15 min of leaching time (NFB-15), the ratio decreased to 0.36 (≈stoichiometric) and remained unchanged for samples NFB-30, NFB-45, and NFB-60. The fast leaching kinetics in the first 5 min of the NFB-HD material’s exposure to the citric acid, followed by moderate kinetics in the 5–15 min time interval, complies with the decrease in the RE/TM ratio, confirming the preferential leaching of the secondary RE-rich phases. Consequently, the leaching rate is reduced after the preferential leaching of the secondary phases is finished. However, the reduced RE/TM ratio (0.35) and RE concentration (25.59 wt.%) measured for sample NFB-120 leached for 120 min indicate that the leaching is selective towards the REs in the matrix phase for such long reaction times. This agrees with the work by Behra et al. [37] and Reisdofer et al. [38] which demonstrated the selective leaching of REs from EoL magnets using organic acids (acetic, citric, and malic), targeting the complete recovery of REs from scrap materials. However, here the leaching of RE-rich phase is of prime interest.

**Figure 5:**
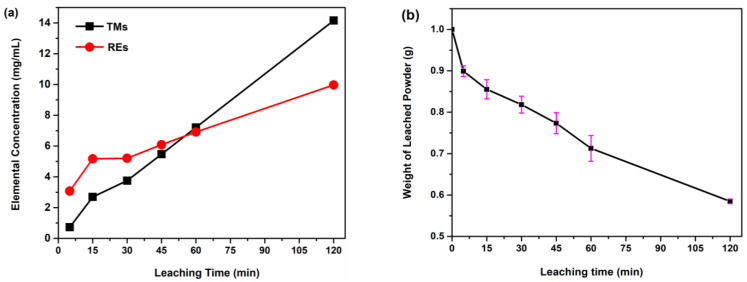
Concentration of TMs (Fe+Co) and REs (Nd+Pr) in the leachates (**a**); the trend of weight loss for NFB-HD powder leached with 1 M citric acid for different leaching times (**b**).

To further investigate the leaching dynamics at leaching times exceeding 15 min, the concentrations of TMs and REs in the leachates corresponding to the different leaching times were considered. Table 3 contains the ICP-MS results revealing the chemical compositions of the leachates after leaching the NFB-HD powder for 5–120 min. The concentrations of both TMs and REs in the leachate increase with the leaching time and do not saturate, indicating that the leaching is continuous. Initially, the RE concentration in the leachate of NFB-5 (3.070 mg/mL) is significantly higher than its TM concentration (0.725 mg/mL), corresponding with the preferential leaching of the secondary RE-rich phases by citric acid. Figure 5a shows the trends of TM and RE concentration change in the leachates depending on the reaction time. The RE concentration is higher than the TM concentration for leaching times up to 45 min. For the NFB-60 leachate, the TM concentration (7.212 mg/mL) exceeds that of REs (6.920 mg/mL). For the 120 min of leaching time, the TM content is significantly higher than the RE content (14.129 and 9.970 mg/mL, respectively). This increase in the TM content, relative to the RE content, confirms that the preferential leaching of RE-rich phases is followed by the dissolution of the matrix grains that are rich in TMs (RE/TM ratio of 0.37). Moreover, from a 30–120 min time period, the trends for RE and TM concentration in Figure 5a are nearly linear, reaffirming that the leaching process is continuous. This is further supported by the weight loss trend of the NFB-HD powder for different leaching times (Figure 5b). After leaching for 15 min (sample NFB-15), which is the period corresponding to the preferential leaching of the RE-rich phases, the weight of the powder was decreased by 15%. This weight loss compares well with the approximate weight fraction of the secondary phases in sintered Nd-Fe-B magnets [22,39]. However, after 120 min of leaching time, the weight was reduced by 41%, reaffirming the leaching of the matrix grains. 

### 3.5. Analysis of Magnetic Properties

**Figure 6:**
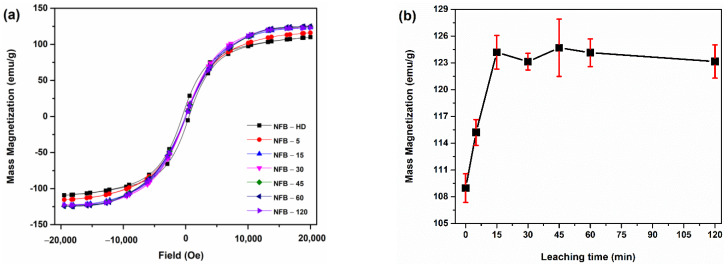
Magnetic hysteresis loops of powder samples (**a**); the dependence of mass magnetization on leaching time (**b**).

The magnetic hysteresis loops of the hydrogen-decrepitated NFB-HD powder and acid-leached powder samples are represented in Figure 6a. The mass magnetization values vs. the leaching time are gathered in Figure 6b. All the samples are magnetically soft with a coercivity of ≈32 Oe for NFB-HD, while the leached powders had a coercivity of ≈3 Oe (Figure 6a). The mass magnetization values increased nearly linearly within the 0–15 min of reaction time: from 110 ± 2 emu/g for the non-leached NFB-HD powder to 115 ± 1 and 124 ± 2 emu/g for samples NFB-5 and NFB-15, respectively. The mass magnetization saturates at ≈124 emu/g for the samples prepared at leaching times of 15 min or longer. The increase in mass magnetization corresponds with the increased volume fraction of the hard-magnetic RE_2_Fe_14_B matrix phase. For sample NFB-5, the SEM (Figure 2b) and compositional analysis (Section 3.4.) revealed a reduced amount of the secondary RE-rich phases compared to the NFB-HD powder; however, the secondary phases were not completely removed during the 5 min of leaching time. Correspondingly, the mass magnetization of sample NFB-5 is below the value measured for the samples leached for longer times. On the other hand, the SEM analysis (Figure 2c), degassing profile (Figure 3), and chemical analysis (Section 3.4) of sample NFB-15 revealed that the removal of the secondary phases was completed within 15 min of the leaching time. The continuous leaching of the matrix grains at longer reaction times, deduced from the compositional analysis of the leachates (Figure 5a) and the powder weight loss accompanying leaching (Figure 5b), does not affect the mass magnetization values. The magnetic characterization results, therefore, confirm that the product of the acid leaching is a single-phase RE_2_Fe_14_B-type powder. 

### 3.6. Discussion on the Leaching Mechanism

According to Marshkov et al. [40], the leaching of a particular phase from the alloy is governed by its electrochemical reduction potential, which depends on the mole fractions of its constituent elements. Therefore, for a chemical approach towards leaching, the selectivity towards a specific phase depends on its reactivity with the leaching agent. A selective acid leaching of secondary phases was recently demonstrated on a fresh Nd-Fe-B powder prepared by conventional means, i.e., strip-casting, hydrogen decrepitation, and jet milling [22]. It was shown that the mild organic triprotic citric acid with dissociation constants 3.13, 4.76, and 6.40 is comparatively more selective towards secondary phases than a strong mineral nitric acid with pKa = −1.5 at room temperature. 

The hydrogenated NFB-HD powder used for the present study can be approximated as a binary system consisting of RE_2_Fe_14_BH_x_ matrix and REH_x_ secondary phase. When exposed to 1 M citric acid, the RE-rich hydride phase is preferentially leached due to the very negative reduction potential of Nd (−2.32 V) and Pr (−2.35 V). The leaching of a RE-rich phase, either in its primary or fully hydrogenated state, proceeds according to the reactions

(2) $$2\mathrm{RE}+3H_{2}O\to {\mathrm{REH}}_{3}+\mathrm{RE}{\left(\mathrm{OH}\right)}_{3}$$

(3) $${\mathrm{REH}}_{3}+3H_{2}O\to \mathrm{RE}{\left(\mathrm{OH}\right)}_{3}+3H_{2}$$

where the RE-rich phase reacts with water molecules, and the evolution of hydrogen gas accompanies the reaction [41]. The citric acid reacts with RE^3+^ from RE(OH)_3_ via chelation, resulting in the formation of soluble metal-ligand RE^3+^- citrate complex (RE_k_H_j_Cit_k_^(3n + j − 3k)^) [42]:

(4) $$\mathrm{nRE}{\left(\mathrm{OH}\right)}_{3}+{\mathrm{jH}}^{+}+{\mathrm{kCit}}^{3-}↔{\mathrm{RE}}_{k}H_{j}{\mathrm{Cit}}_{k}{}^{\left(3n+j-3k\right)}+3n{\left(\mathrm{OH}\right)}^{-}$$

For 5 min of reaction time, acid leaching of the powder resulted in more than four times higher REs concentration in the leachate compared to TMs (3.070 and 0.725 mg/mL, respectively, Table 2). The presence of TMs in the leachate could signify partial leaching of the matrix phase accompanying the preferential leaching of secondary phases. However, TMs are known to be present in the RE-rich secondary phases, particularly at the grain boundaries [36], which makes the origin of TMs in the leachate corresponding to 5 min of reaction time uncertain. Nevertheless, the results of our study show that for the leaching time of 0–15 min, RE-rich phases are preferentially leached, and the matrix grains are recovered from the initially multi-phase, hydrogenated, Nd-Fe-B-type material. Further work is needed to investigate the effect of acid leaching on the structural and chemical composition of the matrix phase, particularly the grains’ surface, which is out of the scope of the present study.

Though the leaching process is continuous, as supported by the experimental results, the loss of the active component in the NFB-HD powder (i.e., the damage to RE_2_Fe_14_B grains) can be avoided by proper optimization of the leaching time as represented in this study.

## 4. Conclusions

Preferential leaching of RE-rich secondary phases by diluted citric acid was explored to extract the RE_2_Fe_14_B matrix grains from sintered Nd-Fe-B-type magnets. Before acid treatment, the magnets were subjected to the hydrogen decrepitation (HD) process to pulverize them and increase the material’s surface-to-volume ratio. The effect of the leaching time on the phase composition and magnetic properties was studied. SEM investigation, thermal degassing profiles, and ICP-MS analyses revealed that preferential leaching of the secondary phases was achieved within 5–15 min of exposure to the 1 M citric acid. Leaching of the RE-rich phases from the magnets’ multi-phase microstructure was governed by the negative reduction potential of Nd and Pr. Once the dissolution of the secondary phases in the acidic medium was finished, continuous leaching of the matrix phase took place without degrading the material’s mass magnetization, reaffirming that the resulting powder is a single-phase RE_2_Fe_14_B-type powder. The quantification of single-phase RE2Fe14B-type powder is well supported by the microstructural studies, degassing profile, compositional analysis by ICP-MS, and magnetic measurements by VSM. The leaching process to extract the hydrogenated RE_2_Fe_14_B grains is compatible with the existing hydrogen processing of magnetic scrap (HPMS), a key technology for the sustainable short-loop recycling of end-of-life sintered Nd-Fe-B-type magnets. Moreover, using a mild organic acid like citric acid makes the leaching process environmentally friendly, considering that the leaching medium is easily neutralized once the reaction is terminated.

## Figures and Tables

**Table 1:** Chemical compositions (at. %) determined via EDS for the locations marked in Figure 1b.

	Al	Fe	Co	Ga	Pr	Nd	O
Site 1	/	86.6	/	/	3.2	10.2	/
Site 2	/	58.6	/	6.2	8.5	19.8	6.9
Site 3	2.0	59.3	/	4.3	8.0	19.9	6.5
Site 4	1.5	67.7	/	3.9	5.7	14.3	6.9
Site 5	/	34.3	10.9	/	16.3	32.8	5.7
Site 6	/	26.2	10.2	/	18.0	34.9	10.7
Site 7	/	3.4	/	/	14.0	36.3	46.3

**Table 2:** Elemental composition (wt. %) of NFB-HD powder and leached powder samples obtained by ICP-MS.

Sample	RE_2_Fe_14_B (Reference)	NFB-HD	NFB-5	NFB-15	NFB-30	NFB-45	NFB-60	NFB-120
**Element**								
**B**	0.99	1.02	0.98	0.95	0.95	0.97	1.02	0.97
**Al**	-	0.13	0.15	0.14	0.14	0.15	0.16	0.17
**Fe**	-	67.18	70.41	70.89	71.22	71.45	71.29	71.70
**Co**	-	0.89	0.91	0.85	0.88	0.87	0.89	0.92
**Fe + Co (TM)**	*72.30*	*68.07*	*71.32*	*71.74*	*72.10*	*72.32*	*72.18*	*72.62*
**Cu**	-	0.09	0.09	0.08	0.08	0.09	0.09	0.12
**Ga**	-	0.22	0.18	0.16	0.15	0.14	0.14	0.13
**Pr**	-	7.35	6.38	6.16	6.03	6.12	6.08	5.89
**Nd**	-	22.86	20.85	20.24	20.16	20.16	20.19	19.70
**Nd + Pr (RE)**	*26.70*	*30.21*	*27.23*	*26.40*	*26.19*	*26.28*	*26.27*	*25.59*
**RE/TM**	0.36	0.44	0.38	0.36	0.36	0.36	0.36	0.35

**Table 3:** Composition (in mg/mL) of leachate solutions obtained by ICP-MS.

Sample	NFB-5	NFB-15	NFB-30	NFB-45	NFB-60	NFB-120
**Element**						
**B**	0.008	0.032	0.044	0.068	0.088	0.174
**Al**	0.003	0.008	0.011	0.014	0.019	0.034
**Fe**	0.690	2.620	3.670	5.350	7.070	13.900
**Co**	0.035	0.076	0.087	0.116	0.142	0.229
**TMs (Fe + Co)**	*0.725*	*2.696*	*3.757*	*5.466*	*7.212*	*14.129*
**Cu**	0.006	0.008	0.004	0.003	0.002	0.002
**Ga**	0.032	0.064	0.068	0.079	0.088	0.104
**Pr**	0.900	1.480	1.480	1.700	1.930	2.610
**Nd**	2.170	3.690	3.730	4.390	4.990	7.360
**Nd + Pr (REs)**	*3.070*	*5.170*	*5.210*	*6.090*	*6.920*	*9.970*

## Data Availability

The data presented in this study are available on request from the corresponding author. The data are not publicly available due to privacy reasons.
